# The “identikit” of subject with obesity and COVID-19 vaccine breakthrough

**DOI:** 10.17179/excli2022-4864

**Published:** 2022-04-08

**Authors:** Giovanna Muscogiuri, Luigi Barrea, Ludovica Verde, Claudia Vetrani, Silvia Savastano, Annamaria Colao

**Affiliations:** 1 Dipartimento di Medicina Clinica e Chirurgia, Endocrinology Unit, University Medical School of Naples, Naples, Italy; 2 Centro Italiano per la cura e il Benessere del paziente con Obesità (C.I.B.O), Department of Clinical Medicine and Surgery, Endocrinology Unit, University Medical School of Naples, Naples, Italy; 3 Cattedra Unesco “Educazione alla salute e allo sviluppo sostenibile”, University Medical School of Naples, Naples, Italy; 4 Dipartimento di Scienze Umanistiche, Università Telematica Pegaso, 80143 Napoli, Italy

**Keywords:** COVID-19, SARS-CoV-2, vaccine breakthrough, obesity, type 2 diabetes, hypertension

## Abstract

The mRNA coronavirus disease 2019 (COVID-19) vaccines were highly effective in the prevention of symptomatic COVID-19, hospitalization, severe disease, and death. However, a minority of vaccinated individuals might become infected and experience significant morbidity. Risk factors of COVID-19 vaccine breakthrough in obesity have not been elucidated. Thus, we aimed to portray the subjects with obesity developing COVID-19 vaccine breakthrough despite vaccination. Coronavirus 2019 (COVID-19) mRNA vaccines have been highly effective in preventing symptomatic COVID-19, hospitalization, severe illness and death. However, a minority of vaccinated individuals may become infected and experience considerable morbidity. The risk factors for COVID-19 vaccine breakthrough in obesity have not been elucidated. Therefore, we aimed to depict individuals with obesity who develop COVID-19 vaccine breakthrough despite vaccination. An online questionnaire was distributed to respondents via a snowball sampling method among subjects with obesity belonging to Italian Associations for people living with obesity aged 18 years and above. Two hundred and thirty-five respondents (44.5±14 years; BMI: 33.3±7.2 kg/m^2^) were included in the study. COVID-19 vaccine breakthrough was noted in 34 % of respondents. A higher prevalence of grade III obesity was detected in subjects with COVID-19 vaccine breakthrough compared to subjects that did not (27.5 % *vs* 13.5 %; p=0.014). In addition, a significant lower prevalence of respondents that completed third dose were found in respondents with COVID-19 vaccine breakthrough compared with respondents that did not develop it (33.8 % *vs *72.9 %; p<0.001). After stratifying respondents with COVID-19 vaccine breakthrough according to the completed doses of vaccine, we found that, although no differences were detected in terms of clinical manifestations of COVID-19, there was a significant higher prevalence of type 2 diabetes and hypertension in respondents that completed third doses compared to respondents that completed first and second doses. In conclusion, COVID-19 vaccine breakthrough was more common in subjects with grade III obesity. The presence of type 2 diabetes and hypertension could counteract the immune potentiating effects of vaccine booster against COVID-19.

## Introduction

The global coronavirus disease 2019 (COVID-19) pandemic is currently still spreading (Khan et al., 2020[10]). Pharmacological approaches for the treatment of COVID-19 infection have mostly the target to avoid severe complications of the disease (Vitiello and Ferrara, 2021[23]; Ferrara et al., 2021[8]). Although there are no antivirals specific for the severe acute respiratory syndrome coronavirus 2 (SARS-CoV-2), a promising efficacy has been demonstrated for some antivirals that were authorized for the treatment of other infectious diseases, such as Remdesivir (Ferrara et al., 2021[8]). A glimpse of hope for preventing the spread of pandemic were given by the vaccines that since December 2020 have been administered in a massive way worldwide. An excellent efficacy and safety profile emerged by the epidemiological data post-authorization of the vaccines in use (mRNA or viral vector) (Vitiello et al., 2021[24]). Although rare, emerging reports described breakthrough SARS-CoV-2 infections in fully vaccinated individuals (Juthani et al., 2021[9]). The risk of SARS-CoV-2 infection in relation to peak antibody levels after COVID-19 vaccination also remains to be clarified; there is evidence that a drop in antibody levels in the course of time may increase this risk (Shrotri et al., 2021[20]). In detail, low neutralizing antibody titres and S-specific IgG antibodies have been suggested as early predictors of SARS-CoV-2 infection (Bergwerk et al., 2021[2]) and some conditions more than others may influence IgG levels. In a single-center longitudinal cohort study, 4026 serum samples from 2607 second dose vaccinated health care workers were analyzed, and the lowest IgG levels were consistently associated with male sex, age over 66 years, immunosuppression, type 2 diabetes, hypertension, cardiovascular diseases, and autoimmune disorders (Lustig et al., 2021[11]). In this respect, parameters associated with COVID-19 vaccine breakthrough have been investigated. Obesity and impaired metabolic health are important risk factors for severe COVID-19 (Muscogiuri et al., 2020[13]; Mahrooz et al., 2021[12]). Obesity is known to represent a state of chronic low-grade inflammation that can play a role in the development of metabolic diseases (dyslipidemia, insulin resistance and type 2 diabetes) and alter innate and adaptive immune responses, thus making the immune system more susceptible to infection and weaker to vaccination, antiviral and other antimicrobial drugs (Pugliese et al., 2022[18]; Muscogiuri et al., 2021[13]; Dhurandhar et al., 2015[7]). Thus, it has been hypothesized that obesity as well as obesity-related metabolic diseases may also favor vaccine-breakthrough SARS-CoV-2 infection in fully vaccinated individuals (Watanabe et al., 2022[25]; Stefan et al., 2021[21]; Juthani et al., 2021[9]). Notably, in a single‐center observational study individuals with central obesity (n=86) had lower antibody titers than those without, after two COVID-19 mRNA vaccine inoculations (Pfizer/BioNTech) separated by 3 weeks, which would unfavorably affect their prognosis in the case of vaccine breakthrough (Watanabe et al., 2022[25]). A study carried out in 54 patients that were fully vaccinated against COVID-19 admitted to the Yale New Heaven Health system hospital reported that among the pre-existing comorbidities, overweight, type 2 diabetes and cardiovascular diseases were frequently seen to confer a decreased vaccine effectiveness (Juthani et al., 2021[9]). Similarly, a recent review found metabolic derangement and type 2 diabetes to be associated with an enhanced risk of vaccine breakthrough SARS-CoV-2 infection (Stefan et al., 2021[21]). Thus, the aims of our study were: 1) to investigate the prevalence of COVID-19 vaccine breakthrough in obesity; 2) to identify the risk factors predisposing to COVID-19 vaccine breakthrough in obesity; 3) to report the clinical aspects of subjects with obesity developing COVID-19 vaccine breakthrough.

## Materials and Methods

This cross-sectional study was carried out from December 2021 to January 2022. All search procedures were carried out in accordance with the pertinent guidelines and regulations of the Declaration of Helsinki. A valid informed consent was obtained by all respondents. The target participants were adults aged 18 years and above with obesity (Body mass index (BMI) ≥ 30 kg/m^2^). Given the constraints of using face-to-face methods during an active pandemic, data were collected using the Google Form platform through an online questionnaire. A snowball sampling strategy was employed to spread the online questionnaire through social media (WhatsApp, Facebook). Participants were then also asked to share the link of the questionnaire with people in their social circle. These social media platforms were chosen because of their wide use worldwide.

### Questionnaire on SARS-CoV-2 vaccination status and COVID-19 disease

The questionnaire was developed through a literature review. The survey form consists of 14 questions covering sociodemographic and anthropometric characteristics (Questions 1 to 5), medical illnesses (Questions 6 to 8), information regarding SARS-CoV-2 vaccination status (Questions 9 to 10) and clinical aspects of subjects with obesity developing COVID-19 vaccine breakthrough (Questions 11 to 14). Questions 12-14 were only answered by respondents who responded 'yes' to Question 11 “Did you test positive for COVID-19?” Sociodemographic and anthropometric characteristics include: age in years, gender, weight (kg), height (cm), BMI (kg/m^2^). Medical illnesses included type 2 diabetes, dyslipidemia, and hypertension. Information regarding SARS-CoV-2 vaccination status were the specific vaccine type and the numbers of administered doses. Finally, questions 12-14 addressed the clinical aspects of COVID-19 disease such as the timing of test positive for COVID-19 and clinical aspects (no symptoms, respiratory failure, myalgias, cold, sore throat, headache, loss of smell-taste perception, asthenia, hospitalization). Questions 6, 7, 8 and 11 could be answered in a dichotomous format "yes" and "no". Originally the questionnaire was developed in English and subsequently translated into Italian. A pilot assessment was conducted among the first 30 respondents recruited through snowball sampling in order to ensure the questions were clearly written, easily understood and unambiguous.

### Statistical analysis

Results have been described as mean ± (standard deviation) SD or number (percentage). Differences in the mean ± SD of age, weight, height and BMI according to COVID-19 “yes” or “no” were analyzed using Student's unpaired t-test. The chi-square (χ2) test that was used to assess differences in the frequency distribution of the categorical variables included in this study. SPSS software (PASW version 21.0, SPSS Inc., Chicago, IL, USA) and the MedCalc® package (version 12.3.0 1993-2012 MedCalc Software bvba-MedCalc Software, Mariakerke, Belgium) were used to analyze the collected data.

## Results

A total of 235 respondents (44.5±14 years; BMI 33.3±7.2 kg/m^2^) participated in the study. The majority of respondents were females (79.6 %). Type 2 diabetes affected 4 % of patients, dyslipidemia 19.1 % and hypertension 21.3 %. Vaccination was not chosen in 3.8 % patients, 4.7 % received 1 dose, 31.9 % received 2 doses and 59.6 % received 3 doses. Thirty-four percent of respondents had COVID-19 infection; in particular regarding 41.3 % had COVID-19 infection before the vaccines, 2.5 % after the first dose of vaccine, 38.8 % after the second dose of vaccine and 17.5 % after the third dose of vaccine. Ten percent of the respondents with COVID-19 infection did not report symptoms while the remaining 90 % of respondents developed symptoms. In particular, 17.5 % of respondents developed respiratory failure, 50 % myalgias, 23.8 % cold, 48.8 % sore throat, 58.8 % of headache, 36.7 % loss of smell-taste perception, 45 % asthenia and 6.2 % underwent to hospitalization (3.8 % ordinary hospitalization and 2.4 % intensive hospitalization). A significant higher prevalence of grade III obesity was detected in respondents with experienced COVID-19 vaccine breakthrough (27.5 % *vs* 13.5 %; p= 0.014) compared to respondents that did not. In addition, a significant lower prevalence of respondents that completed third dose were found in respondents with COVID-19 vaccine breakthrough compared with respondents that did not develop it (33.8 % *vs* 72.9 %; p<0.001). After stratifying respondents with breakthrough symptomatic COVID-19 infection according to the completed doses of vaccine, we found that there were no significant differences in terms of age, BMI and clinical manifestations, but there was a significant highest prevalence of type 2 diabetes and hypertension in respondents that completed third doses compared to other 2 groups (see Table 1(Tab. 1)).

## Discussion

COVID-19 vaccine breakthrough is matter of concern and data regarding these infections are still lacking, mostly in subjects with obesity. Vaccines have effectiveness in decreasing risk of getting COVID-19 infections by 70-90 % and shield from severe infections. It has been reported that subjects with obesity are more likely to develop COVID-19 vaccine breakthrough (de Lusignan et al., 2020[6]), and thus, we aimed to identify the identikit of these subjects. Interestingly we found that subjects developing COVID-19 vaccine breakthrough had a higher prevalence of grade III obesity and a lower prevalence of completing third dose of vaccine. In agreement with these data a study carried out by the Oxford Royal College of General Practitioners (RCGP) Research, and Surveillance Center primary care network found that 20.9 % of people with obesity tested positive for the disease compared with only 13.2 % of normal weight people (de Lusignan et al., 2020[6]). In addition, another study carried out in 9386 UK biobank study participants tested for SARS-CoV-2 UK found that the BMI category had a linear association with testing positive for SARS-CoV-2 among participants <65 years (RR=1.09, 95 %, CI 1.02-1.17) (Christensen et al., 2021[4]). Although in normal weight subjects it is expected a decrease of COVID-19 illness severity as the doses of vaccine administered increase (Vitiello et al., 2021[24]), we interestingly found that the COVID-19 illness severity did not change across the groups of respondents that completed the first, second and third doses of vaccine. Although BMI values were comparable among the 3 groups, there was a significant highest prevalence of respondents with type 2 diabetes and hypertension in respondents that developed COVID-19 vaccine breakthrough after completing the third dose. This interesting finding allows us to hypothesize that the coexistence of cardiometabolic comorbidities along with obesity could counteract the immune potentiating effects of vaccine booster against COVID-19. In agreement with our findings, it has been reported a vaccine breakthrough to be more common in subjects with type 2 diabetes and hypertension in a retrospective multicenter cohort study carried out in patients fully vaccinated with Pfizer/BioNTech BNT162b2 vaccine who contracted COVID-19 more than 7 days after the second dose of vaccine and required hospitalization (Brosh-Nissimov et al., 2021[3]). Interestingly, first-dose induced IgG response has been detected to be significantly lower in individuals with type 2 diabetes and/or hypertension and this could contribute to explain the increased risk of experiencing vaccine-breakthrough in subjects with obesity having these comorbidities (Brosh-Nissimov et al., 2021[3]). 

Several reasons could contribute to this, including metabolic alterations that often accompany visceral adiposity, along with immune dysfunction that has been described in patients with obesity (Andersen et al., 2016[1]). In fact, it has been reported that obesity is associated with sub-optimal seroconversion after vaccine administration (Painter et al., 2015[16]) and even if seroconversion seems to be effective, an increased risk of infection has been detected compared with normal weight subjects (Neidich et al., 2017[15]). In fact, a recent clinical trial in 248 Italian health workers has demonstrated that higher BMI is related to lower Ab titres in response to the COVID-19 vaccine (Pellini et al., 2021[17]). 

Also, hyperglycemia may further increase the risk of getting infections, as increased glucose levels have been reported to promote virus replication and cytokine production in monocytes, as well as glycolysis has been found to support monocyte response and viral replication induced by SARS-CoV-2 (Codo et al., 2020[5]). Premature immunosenescence (i.e. early aging of the immune system) has been identified in people with obesity or type 2 diabetes, particularly of the CD4+ and CD8+ T-cell compartments (Stefan et al., 2021[21]). In this regard, since obesity is frequently associated with a state of insulin resistance, it has been observed that impaired insulin signaling plays an important role in modulating the body's immune response. In rodents, T cells lacking the insulin receptor were found to have reduced inflammatory potential and poor protective immunity against H1N15 influenza infection (Tsai et al., 2018[22]). Specifically, then, hyperinsulinemia, that is usually observable in insulin resistance, has been reported to increase the expression of GRP78 in adipocytes as a binding protein spike partner of SARS-CoV-2 and ACE2 (Shin et al., 2021[19]). This effect could induce an increase in the rate of adipocyte infection, resulting in the unfavorable progression of COVID-19 in SARS-CoV-2 infected persons (Shin et al., 2021[19]).

We recognize that there are certain limitations to the present study. First, due to the cross-sectional nature of this study, it was not possible to identify a causal relationship between SARS-CoV-2 vaccination status and obesity. Furthermore, the questionnaire, although easy to perform by the participants, only allows for relative but not absolute statements and being self-reported, there may have been biases related to completion.

In conclusion our study is the first to investigate risk factors predisposing obesity to COVID-19 vaccine breakthrough. Individuals with grade III obesity are at high risk of developing vaccine breakthrough; in addition, the coexistence of type 2 diabetes and/or hypertension along with obesity may contribute to reduce antibody response following the vaccine, suggesting that COVID-19 vaccination strategies should be re-evaluated in these more susceptible populations. Indeed, the treatment of the co-existing comorbidities such as type 2 diabetes and/or hypertension along with weight loss should be promoted in order to potentiate the vaccine immune responses in subjects potentially at risk of COVID-19 vaccine breakthrough.

## Declaration

### Author contributions

Conceptualization: G.M. and L.B.; methodology: G.M.; formal analysis: C.V.; writing-original draft preparation, G.M. and L.B.; writing-review and editing: G.M. and L.V.; visualization, S.S. and A.C.; supervision, S.S. and A.C. All authors have read and agreed to the published version of the manuscript.

### Competing interests

The authors declare no conflict of interest.

### Funding

No funds, grants, or other support were received.

### Financial interest

The authors have no relevant financial or non-financial interests to disclose.

### Ethics approval

All procedures performed in studies involving human participants were in accordance with the ethical standards of the institutional and/or national research committee and with the 1964 Helsinki Declaration and its later amendments or comparable ethical standards. This is an observational study. The Federico II Ethics Committee has confirmed that no ethical approval is required.

### Consent to participate

Informed consent was obtained from all individual participants included in the study. 

### Data availability 

All data generated or analyzed during this study are included in this published article.

## Figures and Tables

**Table 1:**
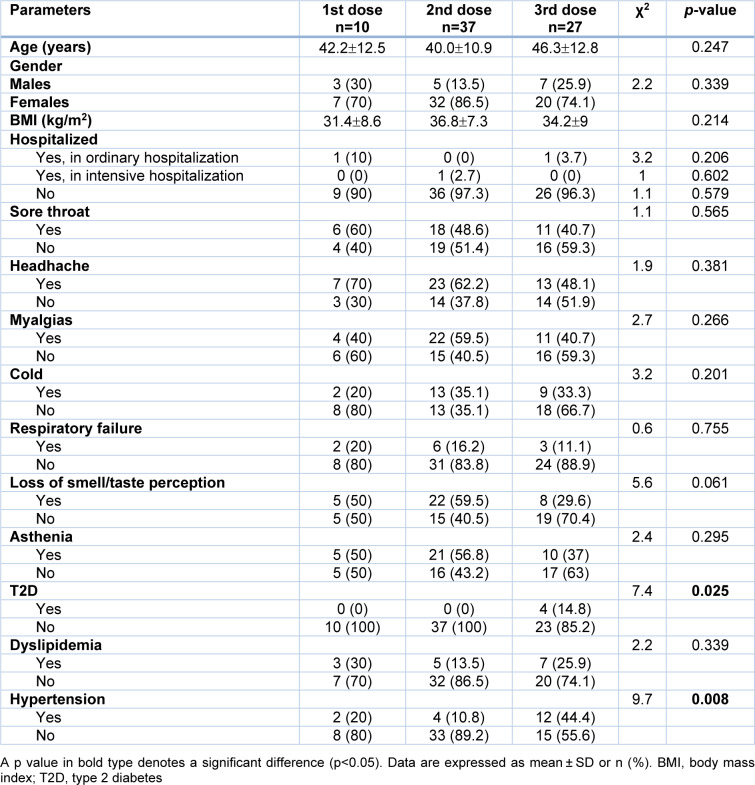
Anthropometric parameters, clinical manifestations, and medical illnesses of COVID-19 vaccine breakthrough according to the completed doses of vaccine
